# Rethinking radiation therapy in the era of RAS inhibition for pancreatic cancer

**DOI:** 10.1093/oncolo/oyag433

**Published:** 2026-10-28

**Authors:** Michael D Chuong, Eileen M O’Reilly

**Affiliations:** Department of Radiation Oncology, Herbert Wertheim Cancer Center, Baptist Health South Florida, Miami, FL 33176, USA; Department of Oncological Sciences, Florida International University Herbert Wertheim College of Medicine, Miami, FL 33199, USA; Gastrointestinal Oncology Service, Department of Medicine, Memorial Sloan Kettering Cancer Center, New York, NY 10065, USA

**Keywords:** pancreatic cancer, RAS inhibition, radiation therapy, stereotactic body radiation therapy, KRAS, daraxonrasib

The recent United States Food and Drug Administration approval of daraxonrasib represents a landmark advance in the treatment of pancreatic ductal adenocarcinoma (PDAC) and raises an important question: will RAS inhibitors diminish the role of local treatments, especially radiation therapy (RT)? The phase 3 RASolute 302 trial demonstrated substantial improvements in overall survival (OS), progression-free survival (PFS), objective response rate (ORR), and quality of life (QOL) with daraxonrasib vs standard chemotherapy in patients with previously treated metastatic PDAC.^1^ The magnitude of benefit was particularly striking in a second-line metastatic population with historically poor outcomes; the OS hazard ratio of 0.40 is especially remarkable when compared to prior studies evaluating other systemic therapies in PDAC.^2^^,^^3^ In addition, median time to deterioration in global health status/QOL was 5.7 months with daraxonrasib vs 2.6 months with chemotherapy (*P* < 0.001) while median time to pain deterioration was 9.2 vs 3.8 months (*P* < 0.001).^1^ As such, attention has naturally pivoted to how RAS inhibition may be used for other PDAC subsets, including patients treated in the first-line metastatic and nonmetastatic settings.

## Preclinical rationale for combining RAS inhibition and radiation therapy

Oncogenic RAS signaling has long been associated with radiation resistance, although a small but growing body of literature demonstrates that RAS inhibition can improve the efficacy of RT in preclinical models of various tumors, including PDAC, through radiosensitization.^4–6^ Laurent et al. showed that the selective KRAS G12C inhibitor MRTX1257 radiosensitized KRAS G12C-mutant murine colon cancer cells in vitro and improved tumor control when combined with radiation in vivo.^4^ Durable responses were notably observed only in immunocompetent mice, suggesting that immune-related effects may be important. In a recent preprint, Wang et al. demonstrated synergistic suppression of pancreatic cancer growth using a combination of stereotactic body radiation therapy (SBRT) and RAS inhibitors MRTX1133 (G12D) and daraxonrasib (multi/pan-RAS(ON)) in vitro and in vivo.^5^ They demonstrated synergy between RT and MRTX1133 in KRAS G12D-mutant cell lines and between RT and daraxonrasib across PDAC cell lines harboring KRAS G12D, G12V, and G12C mutations. In orthotopic pancreatic tumor models, SBRT was prescribed to a clinically relevant dose of 40 Gy in 5 fractions; RAS inhibition was initiated 6 hours before the first fraction and continued during and after SBRT. Median survival was approximately 43 days with SBRT alone, 65 days with RAS inhibition alone, and was significantly prolonged when SBRT was combined with either RAS inhibitor. SBRT plus MRTX1133 achieved complete tumor regression without observed recurrence and the longest survival reached 560 days. SBRT plus daraxonrasib also prolonged survival, although most mice had detectable tumors at euthanasia.^5^ Treatment-induced ferroptosis was implicated in the observed synergy, and the addition of a ferroptosis inhibitor abrogated the improved tumor control and survival benefit of the RT plus KRAS inhibitor combination.

RAS inhibition remodels the tumor microenvironment, providing additional biological rationale for investigating the combination with RT.^7–9^ Kemp et al. demonstrated that MRTX1133 increased collagen deposition and myofibroblasts while promoting macrophage polarization toward a proinflammatory M1-like phenotype.^7^ They also demonstrated that T-cell depletion accelerated tumor regrowth after treatment, suggesting that adaptive immunity contributes to the durability of response. Mahadevan and colleagues similarly reported an important role for T cells in the response to MRTX1133, which increased intratumoral CD8+ effector T cells, decreased myeloid infiltration, and reprogrammed cancer-associated fibroblasts.^8^ Kumarasamy et al. also showed that the efficacy of RAS inhibition was associated with suppression of neutrophils and an influx of CD8+ T cells.^9^

## Clinical rationale for integrating radiation therapy and RAS inhibition

These preclinical data present a compelling argument to explore RAS inhibition and RT in prospective clinical trials, where these treatments may have complementary rather than competing roles in PDAC. Although RASolute 302 established daraxonrasib as a highly effective new standard of care for second-line metastatic PDAC, most patients experience disease progression over time.^1^ Nonetheless, RASolute 302 provides critical evidence that daraxonrasib substantially improves disease control compared to conventional chemotherapy, which may create a greater opportunity for durable local control to be clinically impactful, especially in nonmetastatic PDAC.

Acquired resistance to RAS inhibitors is common and provides a strong rationale for future trials to evaluate combination regimens.^10^^,^^11^ Aronchik et al. identified treatment-emergent RAS signaling pathway alterations in 26 of 44 (59%) patients evaluated at disease progression after treatment with daraxonrasib.^10^ Although resistance mechanisms are not fully understood, both genetic and non-genetic mechanisms have been implicated.^11^ Preclinical data suggest that multi-agent regimens including RAS inhibitors combined with chemotherapy may improve outcomes, supporting ongoing clinical evaluation.^11^ For example, the phase 3 RASolute 303 trial (NCT07491445) is comparing daraxonrasib with or without gemcitabine/nab-paclitaxel against chemotherapy alone in first-line metastatic PDAC. The combination of RAS inhibitors and RT also might be an attractive strategy, since RAS inhibition may provide increasingly effective systemic control, especially of disseminated microscopic disease, while advances in image-guided and adaptive RT offer a complementary strategy to achieve long-term control of gross tumor through the safe delivery of ablative doses.

Prospective single-arm studies have reported encouraging local control with ablative RT. The phase 2 SMART trial evaluated ablative 5-fraction SBRT in 136 patients with borderline resectable or locally advanced PDAC after induction chemotherapy and reported favorable 2-year local control after SBRT of 78.2% while 2-year distant PFS was only 28.5%.^12^^,^^13^ The phase 2 MAIBE trial evaluated 15- or 25-fraction ablative chemoradiation in 48 patients with locally advanced PDAC following induction chemotherapy, and similarly reported that 73% experienced distant progression within 2 years of chemoradiation.^14^ In contrast, 2-year local progression/recurrence was only 11% and 15% in the nonsurgical and surgical cohorts, respectively. Despite distant progression remaining the predominant pattern of disease failure, the SMART and MAIBE trials both reported encouraging 2-year OS of approximately 40% from the start of RT, raising the possibility that more effective systemic disease control may translate into even greater clinical benefit.^13^^,^^14^ Definitive RT is also an option for patients with resectable PDAC who are medically inoperable or decline surgery.^15^^,^^16^ Some of these patients may also be unable or unwilling to receive chemotherapy, providing a rationale for prospective evaluation of RAS inhibition with RT.

The role of ablative RT has recently expanded to oligometastatic PDAC, with the 2026 ASTRO pancreatic cancer guideline conditionally recommending definitive RT in selected patients.^16^ The randomized phase 2 EXTEND trial evaluated the addition of metastasis-directed therapy to systemic therapy in patients with oligometastatic PDAC and 5 or fewer metastases.^17^ Local treatment consisted predominantly of stereotactic radiation to all active sites of disease, including the primary tumor when present. The addition of local therapy significantly improved median PFS from 2.5 to 10.3 months compared to systemic therapy alone. Median time to next-line systemic therapy was longer with local therapy (19 vs 8 months), although this difference was not statistically significant.^17^ These findings support the potential benefit of combining durable local control of gross disease achieved with ablative RT with more effective systemic therapy to delay progression at untreated sites. Given the activity of daraxonrasib in metastatic PDAC, prospective evaluation of its integration with ablative RT in oligometastatic disease is warranted.

## Safety considerations and future directions

Although combination strategies may be attractive, RAS inhibitors may have overlapping toxicities with abdominal RT, and it is not known whether they radiosensitize normal tissues. This is an important consideration given that gastrointestinal toxicity was common with daraxonrasib in RASolute 302, including treatment-related diarrhea in nearly 60% of patients along with other mucosal injury.^1^ Some mutant-specific KRAS inhibitors may have a more favorable gastrointestinal toxicity profile than multi/pan-RAS(ON) inhibitors, which may influence the feasibility of combination approaches with RT.^18^ Contemporary prospective ablative RT trials have demonstrated that ablative doses can be safely delivered with low rates of severe treatment-related gastrointestinal adverse events, but these findings do not establish the safety of concurrent RAS inhibition.^12^^,^^13^ Although preclinical studies suggest that RAS inhibition may enhance radiation sensitivity, these findings require clinical validation. The effects of RAS inhibition on cellular proliferation and radiation response are complex, and the interactions observed in experimental models may not fully translate to humans. The safety of concurrent daraxonrasib and radiation therapy has not been established, and prospective phase I studies are needed to evaluate the safety and tolerability of concurrent treatment and also inform optimal treatment sequencing.

RASolute 302 has fundamentally changed the landscape of PDAC and provides a framework on which future therapeutic strategies are being built. The magnitude of benefit achieved with daraxonrasib provides a compelling rationale for prospective trials to define optimal combination strategies with RAS inhibitors and determine how these agents should be integrated into multidisciplinary treatment pathways that incorporate RT. Rather than reducing the role of local therapy, increasingly effective systemic disease control achieved with RAS inhibitors and other novel agents may create a greater opportunity for durable local control to translate into improved long-term outcomes across the spectrum of PDAC.

## Data Availability

No new data were generated or analyzed in support of this article.

## Originality statement

This manuscript is original, has not been published previously, and is not under consideration for publication elsewhere.
